# High Performance Asymmetric Supercapacitor Based on Hierarchical Carbon Cloth In Situ Deposited with h-WO_3_ Nanobelts as Negative Electrode and Carbon Nanotubes as Positive Electrode

**DOI:** 10.3390/mi12101195

**Published:** 2021-09-30

**Authors:** Jianhao Lin, Xusheng Du

**Affiliations:** Institute of Advanced Wear & Corrosion Resistant and Functional Materials, Jinan University, Guangzhou 510632, China; linforvo@163.com

**Keywords:** flame catalytic deposition, WO_3_ nanobelt, CNT, redox-active electrolyte, asymmetric supercapacitor

## Abstract

Urchin-like tungsten oxide (WO_3_) microspheres self-assembled with nanobelts are deposited on the surface of the hydrophilic carbon cloth (CC) current collector via hydrothermal reaction. The WO_3_ nanobelts in the urchin-like microspheres are in the hexagonal crystalline phase, and their widths are around 30–50 nm. The resulted hierarchical WO_3_/CC electrode exhibits a capacitance of 3400 mF/cm^2^ in H_2_SO_4_ electrolyte in the voltage window of −0.5~0.2 V, which makes it an excellent negative electrode for asymmetric supercapacitors. To improve the capacitive performance of the positive electrode and make it comparable with that of the WO_3_/CC electrode, both the electrode material and the electrolyte have been carefully designed and prepared. Therefore, the hydrophilic CC is further coated with carbon nanotubes (CNTs) to create a hierarchical CNT/CC electrode via a convenient flame synthesis method, and a redox-active electrolyte containing an Fe^2+^/Fe ^3+^ couple is introduced into the half-cell system as well. As a result, the high performance of the asymmetric supercapacitor assembled with both the asymmetric electrodes and electrolytes has been realized. It exhibits remarkable energy density as large as 403 μW h/cm^2^ at 15 mW/cm^2^ and excellent cyclic stability after 10,000 cycles.

## 1. Introduction

Although supercapacitors have been considered a new type of energy storage device because of their long cycle life, fast charge and discharge, and excellent power density [1,2], their low energy density severely limits their practical applications. Many researchers have been devoted to increasing the energy density of supercapacitors while ensuring a considerable power density [3,4,5]. Designing and assembling new asymmetric supercapacitors are effective methods to expand their potential and increase their energy density via the integration of various electrode materials and electrolytes [6,7].

It is well known that supercapacitors can be classified into two categories according to the principle of energy storage: electric double-layer capacitors and pseudo-capacitors. In comparison with the former, pseudo-capacitors store energy through a reversible oxidation-reduction reaction during the charging and discharging process, which can provide a larger specific capacitance. Transition metal oxides and hydroxides have been extensively studied as electrode materials in pseudo-capacitors [8,9,10]. As a typical metal oxide, WO_3_ has multiple crystal phases and oxidation states (W^2+^~W^6+^), high theoretical specific capacitance (~1112 F/g), and good electrochemical stability in an acid electrolyte, and it has been demonstrated to be an excellent electrode material [11,12]. Monoclinic, tetragonal, hexagonal, and orthorhombic WO_3_ have been synthesized by adjusting the reaction temperature and pH of the precursor solution, and it has been revealed that the material with the hexagonal phase is the best one for capacitors [13]. Besides the crystal phase, the nanostructure of the metal oxide has also been found to have a strong influence on their properties. Tungsten oxide with various structures has been fabricated and studied, including the one-dimensional (1D) nanorods [14], the two-dimensional (2D) nanoplates [15], and the three-dimensional (3D) nano/microspheres or nanoflowers [11,16,17,18,19]. Specifically, 3D tungsten oxide assembled with building blocks in nanoscale is suggested to be a superior electrode material as it can provide more active sites with considerably larger specific surface areas and buffer the physical strain and stress generated during charging and discharging cycles [16]. For instance, pure WO_3_ nanoflowers in H_2_SO_4_ displayed a capacitance of 127 F/g, and it was greatly enhanced to be 495 F/g after being coated with reduced graphene oxide [11]. The urchin-like tungsten oxide made of WO_2.72_ nanowires exhibits a capacitance of ~235 F/g at 20 A/g in H_2_SO_4_ electrolyte [19]. Generally speaking, 3D nanostructures could also facilitate the mass transport of the electrochemically active species in the electrode/electrolyte interface and promote the performance of the electrode materials. Therefore, materials with such hierarchical structures are attracting more and more interest from researchers in the field of energy conversion and storage devices [20,21,22].

Apart from transition metal oxide electrodes that display high electrochemical activity in the negative voltage range and can be used as a perfect negative electrode in asymmetric supercapacitors, the positive electrode needs to be carefully selected and designed to assemble a high-performance energy storage device. Carbon-based electrode materials have been widely used in these devices. Compared with pseudo-capacitance electrode materials, they have better physical and chemical stability, higher electrical conductivity, larger specific surface area [23,24,25], and more importantly, stable electrochemical performance in acid electrolytes in the wide potential window. However, the low capacitance of the carbon-based materials may limit their application in supercapacitors [26,27]. Recently, being coupled with the redox-active electrolyte has been identified as an effective way to improve their electrochemical performance as the occurrence of the redox reaction of the additives in the electrolyte on the electrode/electrolyte interface will provide additional pseudo-capacitance for the electrochemical system [28]. In fact, adding redox-active Fe^2+/3+^ into the acid electrolyte to promote the capacitive performance of the composite electrodes has been demonstrated in our previous work [28]. The common carbon-based current collector, such as carbon cloth, has the disadvantages of poor hydrophilicity and insufficient surface activity [29], which makes it difficult to be directly used as the electrode in the aqueous electrolytes. Therefore, it is worthwhile to develop effective methods to improve the surface state and the performance of the carbon cloth for its utilization in supercapacitors.

Herein, urchin-like WO_3_ microspheres made of nanobelts were in situ deposited onto carbon cloth, and the as-produced electrode was directly used as the negative electrode for assembling an asymmetric supercapacitor. Meanwhile, the surface of the carbon cloth was also modified, and CNTs were grown on carbon fibers via a convenient flame deposition method to build a hierarchical electrode, which was functionalized with organic groups. The resultant hierarchical electrode was used as the positive electrode. In the meantime, a redox-active electrolyte containing iron ions was introduced into the electrolyte to promote the performance of the half-cell system. The effect of the surface modification (as described above) of the carbon cloth on its electrochemical behavior in the redox-active electrolyte will be comprehensively studied. The performance of the asymmetric supercapacitors assembled with both the hierarchical electrodes and electrolytes will be investigated as well.

## 2. Materials and Methods

### 2.1. Preparation of WO_3_/CC

Before the deposition of WO_3_, carbon cloth was subjected to hydrophilic treatment according to a method reported recently [30]. In detail, carbon cloth (CC, W0S1009) with a size of 4 × 6 cm^2^ was ultrasonically cleaned with acetone, alcohol, and deionized water in sequence. After being dried in an oven at 60 °C, it was submerged in a mixed solution of 10 mL 98 wt% H_2_SO_4_ and 30 mL 68 wt% HNO_3_ and transferred to a 100 mL Teflon-lined stainless-steel autoclave. The autoclave was sealed and heated at 90 °C for 6 h. After being cooled to room temperature, the CC was taken out and ultrasonically cleaned with deionized water to remove the residual acid and then put into an oven at 60 °C.

For the growth of WO_3_ onto the CC, 2.5 mmol of Na_2_WO_4_·2 H_2_O was added to 30 mL deionized water and then stirred until it was completely dissolved. The pH of the solution was adjusted to 1.2 with 3 M HCl. An amount of 7 mmol of oxalic acid was subsequently added to the solution, which was further diluted with deionized water to 50 mL to obtain a WO_3_ sol. After being added to 2 g of (NH_4_)_2_SO_4_, it was transferred to a 100 mL Teflon-lined stainless-steel autoclave along with the hydrophilic-treated CC. The autoclave was sealed and heated at 180 °C for 16 h. After being cooled down naturally to room temperature, the product was taken out and washed with alcohol and deionized water. Finally, the product was dried in an oven at 60 °C. The as-prepared product was named “WO_3_/CC”.

### 2.2. Preparation of CNT/CC

The hydrophilic-treated CC was soaked in a 1 M Ni(NO_3_)_2_ alcohol solution. After the evaporation of the solvent, the sample was inserted into an alcohol flame for 5 min. The temperature of the sample in the flame was measured at 700 °C. The product was named “CNT/CC”. For the convenience of comparison, the carbon cloth subjected to the hydrophilic treatment was named “CC”, and the pristine carbon cloth that had not been subjected to the hydrophilic treatment was named “PCC”.

### 2.3. Assembly of the Asymmetric Supercapacitor

**Figure 1:**
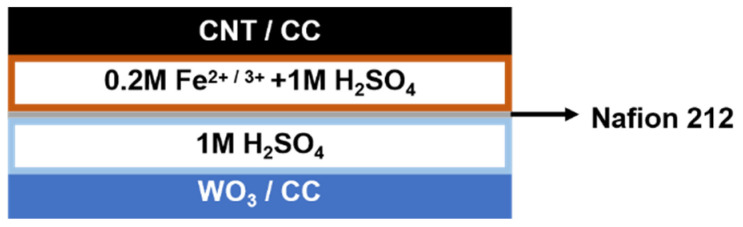
Schematic diagram illustrating the assembly of the ASC device.

The as-prepared CNT/CC and WO_3_/CC with a size of 3 × 8 mm^2^ were used directly as electrodes to assemble the asymmetric capacitor, where the positive half-cell compartment was the CNT/CC electrode in 0.2 M Fe^2+/3+^ + 1 M H_2_SO_4_ electrolyte, and the negative one was the WO_3_/CC electrode in 1 M H_2_SO_4_ electrolyte. The two different half-cell systems were separated by a Nafion 212 proton-exchange membrane. The resultant ASC device with a configuration of CNT/CC/0.2 M Fe^2+/3+^ + 1 M H_2_SO_4_//1 M H_2_SO_4_/WO_3_/CC is shown in Figure 1.

### 2.4. Characterizations

A scanning electron microscope (SEM, Phenom XL, PHENOMSCIENTIFIC, Shanghai, China) and an energy-dispersive spectrometer (EDS, Phenom XL, PHENOMSCIENTIFIC, Shanghai, China) were used to characterize the morphology and the element distribution of the sample, respectively. A transmission electron microscope (TEM, JEM-2100F, JEOL, Tokyo, Japan) was also used to characterize the morphology and crystal structure of the samples. An X-ray diffraction (XRD, UItima IV, Rigaku Corporation, Tokyo, Japan) pattern with a scanning angle ranging from 20° to 80° at a rate of 5°/min was employed to analyze the crystal phase of the sample. A Fourier transform infrared spectrometer (FTIR, VERTEX70, Bruke, Germany) with a wavenumber range from 800 to 2000 cm^−1^ was used to characterize the functional groups on the surface of the sample.

The cyclic voltammetry (CV), the galvanostatic charging/discharging (GCD), and the electrochemical impedance spectroscopy (EIS) tests were performed on the electrodes on an electrochemical workstation (CHI760e, CH Instruments, Shanghai, China). First, a standard three-electrode test system was used to evaluate the properties of the CNT/CC and the WO_3_/CC electrodes individually with a saturated calomel electrode (SCE) as a reference electrode and a Pt plate as the counter electrode. The EIS was measured in the frequency range of 0.01–100 kHz at the open-circuit voltage with an amplitude of 5 mV. The areal-specific capacitance (C, mF/cm^2^), areal energy density (E, mW h/cm^2^), and power density (*p*, mW/cm^2^) were calculated from the following equations: C = I × t/(s × V), E = C × V^2^/(2 × 3.6), and *p* = (E × 3.6)/t, respectively, where I is the discharge current (A), t is the discharge time (s), V is the potential window (V), and s is the effective area (cm^2^) of the electrode of the device.

## 3. Results and Discussions

### 3.1. The Structure and Electrochemical Behavior of the WO_3_/CC Electrode

**Figure 2:**
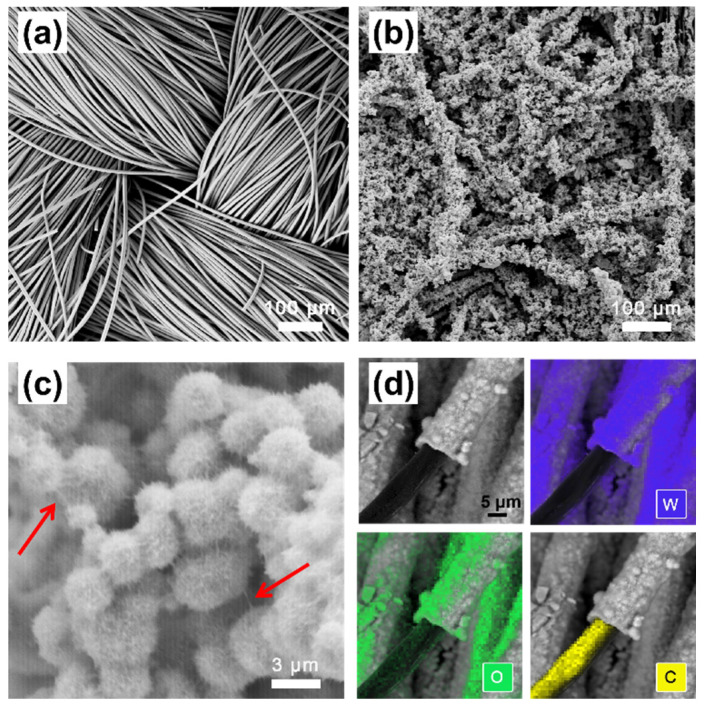
SEM images of (**a**) CC; (**b**,**c**) WO_3_/CC; and (**d**) element distribution of W, O, and C on the WO_3_/CC electrode.

The carbon cloth is woven with carbon fibers. As shown in Figure 2a,b after the hydrothermal reaction, the surface of the CC becomes much rougher, and granular products can be observed to be evenly distributed all over the carbon fibers of the CC. Specifically, most WO_3_ particles are urchin-like microspheres with an average diameter of ~3.5 μm (Figure 2c). The element mapping of an individual fiber of the WO_3_/CC electrode manifests the location of the WO_3_. As shown in Figure 2d, the yellow layer represents the C element while the purple and green layers represent the elements W and O, respectively, indicating that the WO_3_ can be deposited evenly around the carbon fibers.

**Figure 3:**
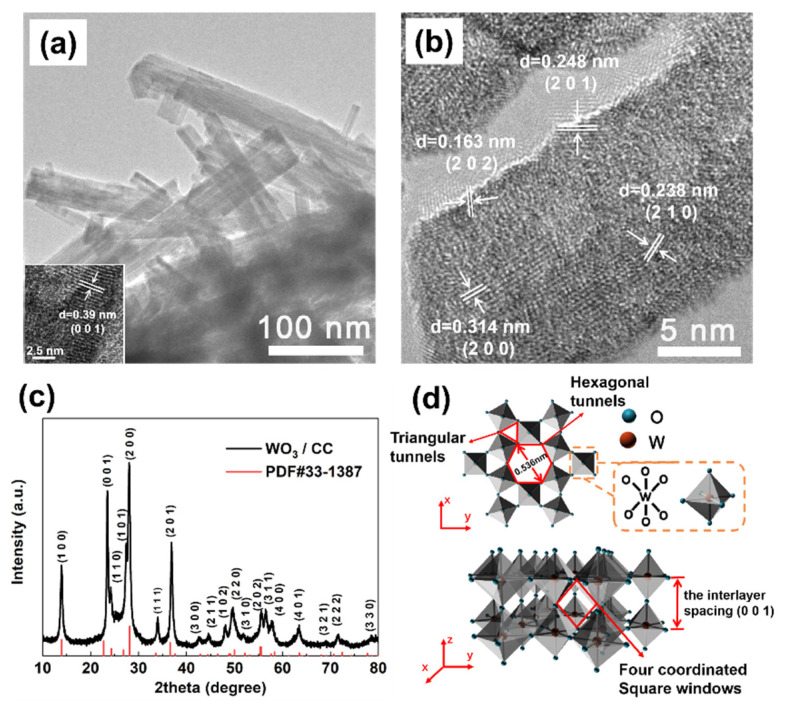
(**a**,**b**) TEM images of WO_3_; (**c**) XRD pattern of WO_3_/CC; and (**d**) the schematic crystal structure of hexagonal WO_3_.

Furthermore, some needles with different lengths can be observed on the rough surface of the WO_3_ microspheres (Figure 2b,c). A TEM analysis was conducted to characterize the structure of the needles in the urchin-like WO_3_ microspheres. As shown in Figure 3a, such needles are actually WO_3_ nanobelts, which were self-assembled into microspheres during the hydrothermal deposition of the WO_3_ onto the CC. The WO_3_ nanobelts in urchin-like microspheres have an average width around 30~50 nm. Moreover, the length of the belts could be as large as several micrometers, as seen from those bridging between the microspheres in Figure 2c, which are highlighted by red arrows. The crystal structure of the WO_3_ was further verified by an XRD. As shown in Figure 3c, all the diffraction peaks of the sample can be indexed to the hexagonal phase of the WO_3_ (h-WO_3_, JCPDS No.33-1387). Moreover, the ordered lattice stripes with spaces of 0.39 nm, 0.314 nm, 0.248 nm, 0.238 nm, and 0.163 nm in the high-resolution TEM (HRTEM) image, as shown in Figure 2a,b, can be assigned to the (001), (200), (201), (210), and (202) planes of the hexagonal WO_3_, respectively, which is also consistent with its XRD analysis, confirming the deposit of the hexagonal phase of the WO_3_ nanobelts on the CC. Figure 3d illustrates the schematic crystal structure of the hexagonal WO_3_ with its layered structure, which is composed of the cubic perovskite-like structure with a (WO_6_) octahedron as the constituent unit. The W atom in the unit is located at the center of the octahedron, while the O atom is located at each vertex of the octahedron, as shown in the orange dotted box in Figure 3d. Three types of tunnels, including triangular and hexagonal types as well as four coordinated square windows (highlighted with red arrows in Figure 3d), are formed in the hexagonal WO_3_ structure based on the rotation of the cubic unit so that the tunnels formed by W-O enable the proton insertion/de-insertion into the crystalline structure, which is beneficial to its electrochemical process in the acidic electrolyte. Additionally, the surface terminal oxygen atom (-O site) is expected to be more actively involved in the redox reactions, which can be reduced to a -OH terminal in the electrolyte. Furthermore, the hierarchical 3D urchin-like WO_3_ microspheres directly grown on the surface of the carbon fibers in the carbon cloth will support fast ion diffusion, improved electrolyte wettability, and the accommodation of large volume expansion during the cyclic test [31].

**Figure 4:**
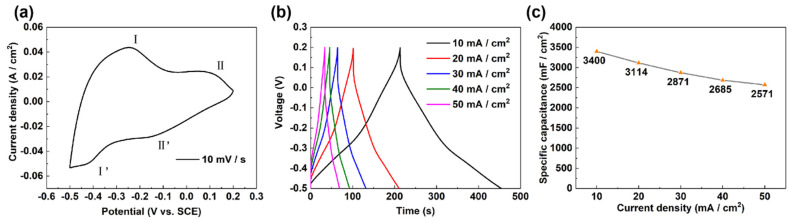
Electrochemical performance of WO_3_/CC in 1 M H_2_SO_4_ electrolyte. (**a**) CV curves at 10 mV/s; (**b**) GCD curve at different current densities; and (**c**) specific capacitance at different current densities.

The electrochemical performance of the WO_3_/CC electrode was evaluated by both CV and GCD tests with a three-electrode system. Figure 4a shows the CV curve of WO_3_/CC at 10 mV/s, where two pairs of reversible redox peaks appear at −0.24 V (peak I), 0.08 V (peak II), −0.17 V (peak II’), and −0.43 V (peak I’). These peaks can be attributed to the two-step electrochemical redox-reaction process of the WO_3_, including the proton diffusion kinetics in the layered structure of the WO_3_ [32,33]. The electrochemical reaction involved in the process can be assigned as: $WO_{3}+xH^{+}+xe^{-}↔H_{X}WO_{3}$. The GCD curves of WO_3_/CC at different current densities were shown in Figure 4b, and the corresponding specific capacitance values were calculated and plotted in Figure 4c. The specific capacitance of WO_3_/CC was 3400 mF/cm^2^ at 10 mA/cm^2^, and it remained at 2571 mF/cm^2^ at 50 mA/cm^2^. The high capacitive performance of the electrode can be attributed to the great affinity of the WO_3_ to the hydrophilic CC, leading to the strong adhesion of the WO_3_ nanobelts to the CC current collector. Therefore, the as-prepared WO_3_/CC can be used as an excellent negative electrode in asymmetric supercapacitors.

### 3.2. The Structure and Electrochemical Performance of CNT/CC

**Figure 5:**
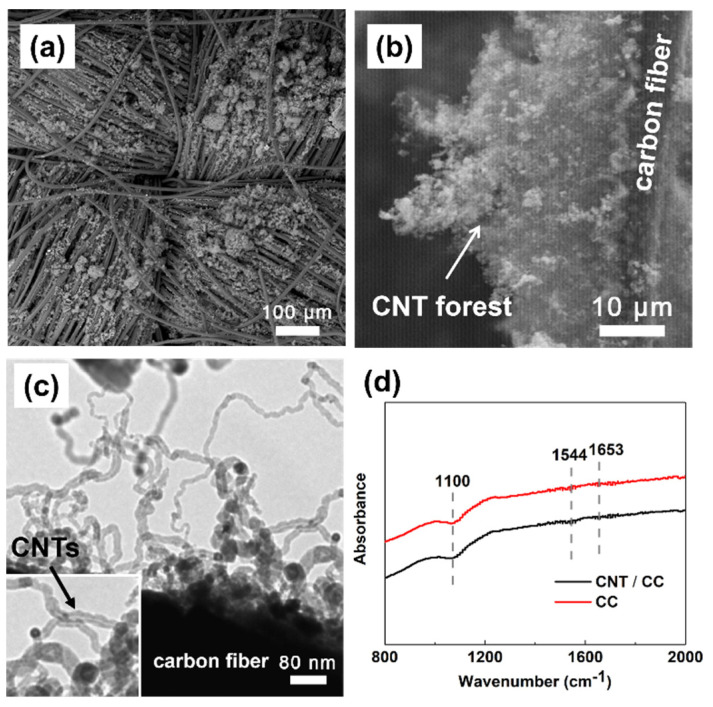
(**a**,**b**) SEM and (**c**) TEM images of CNT/CC; and (**d**) FTIR spectrum of CC and CNT/CC.

The structure of the CNT/CC electrode was analyzed by SEM. Compared with the smooth surface of the CC, as shown in Figure 2a, the fluffy surface of the CC with a cluster structure distributed evenly can be observed after the flame treatment (Figure 5a). In the SEM image at high magnification (Figure 5b), it can be observed that the clusters on the CC are actually CNT agglomerates, where a CNT forest has grown on a single carbon fiber. This result is different from the fine, single CNT that had been deposited inside the thicker carbon nanotubes with a similar flame method as was reported recently [34]. The possible reason could be the limited nanospace of the tube tunnel for the flame growth of CNTs. The image from the TEM in Figure 5c shows the hollow structure of the deposited CNTs. Different from most commercial CNTs fabricated with the CVD method, the flame-synthesized CNTs appear to be wavy rather than straight. They have a diameter of 10~40 nm and have grown randomly and entangled with each other on the surface of the carbon fiber, as shown in Figure 5b,c. Furthermore, although the hierarchical carbon materials display weak FTIRATR signals and slanted baselines as usual, as shown in Figure 5d, the detected peaks at 1100, 1544, and 1653 cm^−1^ can be attributed to the C-O, C-OR, C=O, and COOH groups, respectively [35], indicating the existence of some organic functional groups after the hydrophilic and flame treatment of the carbon cloth. As revealed before, the flame-synthesized 1D carbon nanomaterials inherently have been modified with oxygen-containing functional groups, which is one of their advantages as electrode materials compared to those produced with CVD methods. Obviously, both the hierarchical structure and the functional carbon components in the as-produced CNT/CC electrode are beneficial to its electrochemical performance.

**Figure 6:**
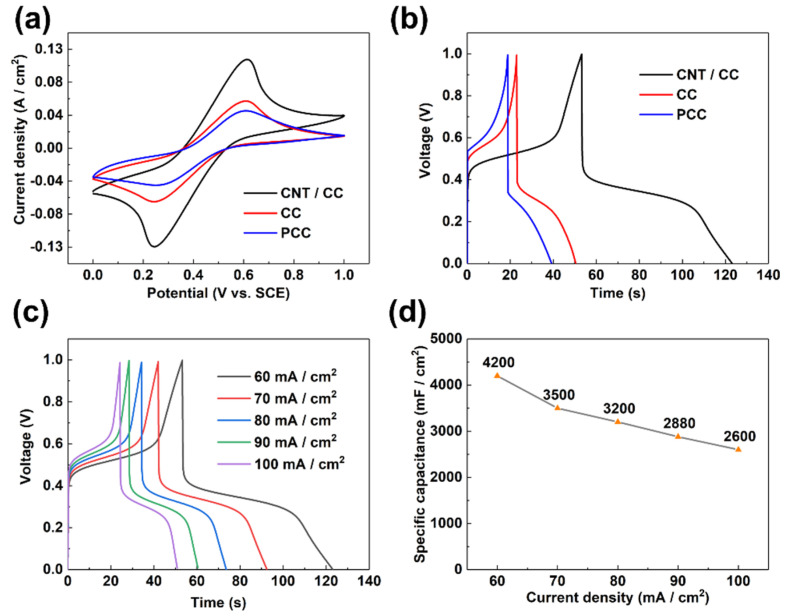
The electrochemical behavior of PCC, CC, and CNT/CC electrodes in 0.2 M Fe^2+/3+^ + 1 M H_2_SO_4_ electrolyte. (**a**) CV curves of PCC, CC, and CNT/CC electrodes at 10 mV/s; (**b**) GCD curves of PCC, CC, and CNT/CC electrodes at 60 mA/cm^2^; (**c**) GCD curves of CNT/CC electrode at various current densities; and (**d**) the specific capacitance of CNT/CC electrode at various current densities.

In order to study the effect of the hydrophilic treatment and CNT decoration on the promotion of the performance of the CC electrode, the electrochemical behavior of the PCC, CC, and CNT/CC has been measured at 0.2 M Fe^2+/3+^ + 1 M H_2_SO_4_ electrolyte under a three-electrode system, respectively. Figure 6a shows that all the CV curves of the three different samples tested at the same condition display a pair of redox peaks at almost the same peak potential, which originates from the electrochemical reaction of the redox-active couples in the electrolyte: $Fe^{3+}+e^{-}↔Fe^{2+}$. In addition, the area of the CV curve of the hydrophilic carbon cloth (CC) is obviously larger than that of the pristine one (PCC). Moreover, after further flame treatment, the resultant CNT/CC sample shows a much larger CV area than both CC and PCC, confirming the superior performance of this hierarchical electrode. The specific capacitance calculated from the GCD curves in Figure 6b is: C _(CNT/CC)_ = 4200 mF/cm^2^ > C _(CC)_ = 1620 mF/cm^2^ > C _(PCC)_ = 1260 mF/cm^2^, indicating that the CNT/CC has the largest specific capacitance out of all three electrodes. This result could be due to the CNTs grown on the carbon fiber surface, which could greatly enlarge the specific surface area of the electrode and provide more active sites for the redox reaction of the iron ion couples, thus leading to a much larger pseudo-capacitance of the electrode. Figure 6c shows the GCD curves of the CNT/CC recorded at different current densities. The corresponding specific capacitance values are shown in Figure 6d. The specific capacitance of the half-cell system at 60 mA/cm^2^ is 4200 mF/cm^2^. When the current density is increased to 100 mA/cm^2^, its specific capacitance still maintains 2600 mF/cm^2^.

### 3.3. The Electrochemical Performance of the Assembled Asymmetric Supercapacitor

**Figure 7:**
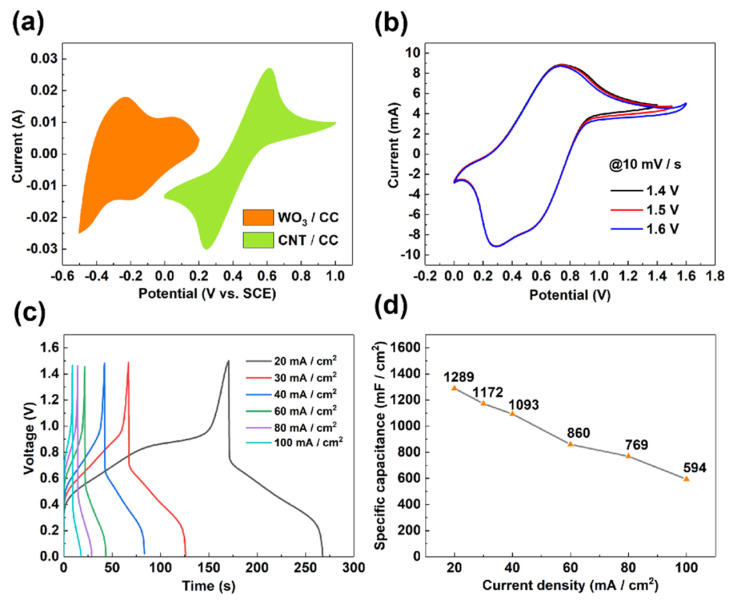
Electrochemical behavior of the ASC device with the configuration of CNT/CC/0.2 M Fe^2+/3+^ + 1 M H_2_SO_4_//1 M H_2_SO_4/_WO_3_/CC. (**a**) CV curves of CNT/CC electrode in 0.2M Fe^2+/3+^ + 1 M H_2_SO_4_ electrolyte and WO_3_/CC electrode in 1 M H_2_SO_4_ electrolyte at 10 mV/s; (**b**) CV curves of the ASC operated in different voltage windows; (**c**) GCD curves of the ASC at different current densities; and (**d**) the specific capacitance of the ASC under different current densities.

Since both the half-cell system of the WO_3_/CC in H_2_SO_4_ and the CNT/CC in 0.2 M Fe^2+/3+^ + 1 M H_2_SO_4_ exhibited high electrochemical performance, it is highly expected that the asymmetric supercapacitors assembled with them will be an energy storage device with a high energy density. In this study, the configuration of the assembled asymmetric supercapacitor can be expressed as CNT/CC/0.2 M Fe^2+/3+^ + 1 M H_2_SO_4_//1 M H_2_SO_4_/WO_3_/CC. As shown in Figure 1, both the electrodes can be well stabilized in their respective electrolytes, and their electrochemical performance will be maximized in the newly designed device. Figure 7a shows the CV curve of the WO_3_/CC and the CNT/CC under their respective “electrode–electrolyte” systems. The areas of the CV curves of the WO_3_/CC and the CNT/CC are almost the same, indicating the charge between the positive and the negative parts in the ASC device is well balanced. Figure 7b shows the CV curve of the ASC under different voltage ranges, which implies that the voltage range of the device is better to be set up as 0~1.5 V, as an obvious polarization can be found when the high potential is larger than 1.5 V. In addition, the evident redox peaks can be observed in the CV curves, indicating the pseudo-capacitance behavior of the assembled ASC, which can be ascribed to the redox reaction of the WO_3_/CC negative electrode in the normal H_2_SO_4_ electrolyte and that of the active couple Fe^2+/3+^ on the CNT/CC positive electrode. Figure 7c shows the GCD curves under different current densities, and the corresponding specific capacitance values calculated from the GCD curve are shown in Figure 7d. Specifically, the area-specific capacitance of the ASC is as high as 1289 mF/cm^2^ at a current density of 20 mA/cm^2^, and it still maintains 594 mF/cm^2^ when the current density is increased to 100 mA/cm^2^.

**Figure 8:**
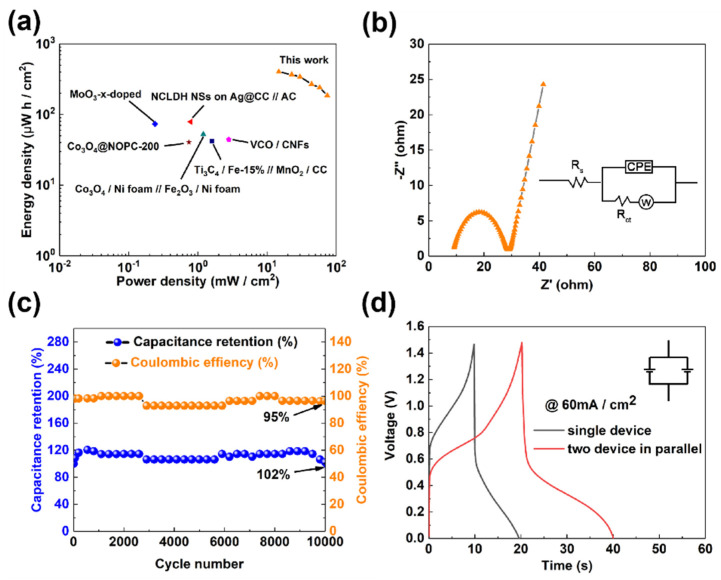
Electrochemical performance of the ASC device in the configuration of CNT/CC/0.2 M Fe^2+/3+^ + 1 M H_2_SO_4_//1 M H_2_SO_4_/WO_3_/CC. (**a**) Ragone plot of ASC device in comparison with other results in literature; (**b**) Nyquist plot of the device; (**c**) multi-cycling test of the device; and (**d**) GCD curves of two devices connected in parallel.

The Ragone plot in Figure 8a displays the energy density and power density of the ASC device, which is calculated from the GCD curves at various current densities in Figure 7c. Significantly, the ASC device exhibits an energy density as high as 403 μW h/cm^2^ (27 m Wh/cm^3^) at a power density of 15 mW/cm^2^ (992 m W/cm^3^), and it still maintains 186 μW h/cm^2^ when the power density is as high as 74 mW/cm^2^. As shown in Figure 8a, the value is much higher than that of other asymmetric supercapacitors reported previously [36,37,38,39,40,41]. The Nyquist plot of the device in Figure 8b shows an equivalent series resistance (Rs = 8.5 Ω), which is lower than the reported result [27], and the measured charge-transfer resistance (R_ct_ = 19.49 Ω) may be caused by the existence of the proton exchange membrane. In addition, the multi-cycling test, shown in Figure 8c, manifests that the ASC device has a capacitance retention rate of 102% after 10,000 cycles. At the same time, its corresponding coulombic efficiency is still as high as 95%, indicating that the device exhibits excellent stability. The remarkable cyclic stability could be due to the hierarchical structure of both the electrodes, which causes the gradual infiltration of the redox-active Fe^3+/2+^ into the entangled CNTs on the CC in the positive half-cell during the long-term cycling process, and the progressively expanding percolation of the small proton into the multi-tunnel crystalline structure of the h-WO_3_ nanobelts in their self-assembled, urchin-like microspheres in the negative part, as shown in Figure 3d. Moreover, after the parallel connection of two ASC devices, the charge and discharge time of the corresponding GCD curve in Figure 8d increases significantly, which proves that our ASC device has the potential for practical application.

## 4. Conclusions

In summary, urchin-like microspheres self-assembled by h-WO_3_ nanobelts with widths around 30~50 nm are deposited on the surface of the hydrophilic CC through a hydrothermal reaction. Due to its excellent specific capacitance (3400 mF/cm^2^ at 10 mA/cm^2^) in 1 M H_2_SO_4_ electrolyte, the resulted hierarchical WO_3_/CC electrode is directly applied as the negative electrode of the ASC device. A simple flame method has been used to deposit CNTs onto the surface of the CC to make the hierarchical positive electrode as well, which exhibits much higher specific capacitance in a redox-active electrolyte than those without the flame treatment. More importantly, the assembled asymmetric supercapacitor device contains both asymmetric electrodes and electrolytes (CNT/CC/0.2 M Fe^2+/3+^ + 1 M H_2_SO_4_//1 M H_2_SO_4_/WO_3_/CC) and exhibits a remarkable energy density as high as 403 μW h/cm^2^ at the power density of 15 mW/cm^2^. Moreover, it maintains excellent long-term cyclic stability after 10,000 cycles, which could be due to the hierarchical structure of both electrodes, including the porous multi-tunnel crystalline structure of the hexagonal WO_3_ nanobelts in the urchin-like microspheres. The novel configuration of the ASC device provides better opportunities for the convenient design and fabrication of the next generation of high-performance supercapacitors.

## References

[1] Saikia B.K., Benoy S.M., Bora M., Tamuly J., Pandey M., Bhattacharya D. (2020). A brief review on supercapacitor energy storage devices and utilization of natural carbon resources as their electrode materials. Fuel.

[2] Sung J., Shin C. (2020). Recent studies on supercapacitors with next-generation structures. Micromachines.

[3] Saeed G., Alam A., Bandyopadhyay P., Lim S., Kim N.H., Lee J.H. (2021). Development of hierarchically structured nanosheet arrays of CuMnO_2_-Mn_x_O_y_@graphene foam as a nanohybrid electrode material for high-performance asymmetric supercapacitor. J. Alloys Compd..

[4] Ouyang Y., Chen Y., Peng J., Yang J., Wu C., Chang B., Guo X., Chen G., Luo Z., Wang X. (2021). Nickel sulfide/activated carbon nanotubes nanocomposites as advanced electrode of high-performance aqueous asymmetric supercapacitors. J. Alloys Compd..

[5] Zhu Z., Gao F., Zhang Z., Zhuang Q., Yu H., Huang Y., Liu Q., Fu M. (2021). Synthesis of the cathode and anode materials from discarded surgical masks for high-performance asymmetric supercapacitors. J Colloid Interface Sci..

[6] Sajjad M., Khan M.I., Cheng F., Lu W. (2021). A review on selection criteria of aqueous electrolytes performance evaluation for advanced asymmetric supercapacitors. J. Energy Storage.

[7] Choudhary N., Li C., Moore J., Nagaiah N., Zhai L., Jung Y., Thomas J. (2017). Asymmetric supercapacitor electrodes and devices. Adv. Mater..

[8] Delbari S.A., Ghadimi L.S., Hadi R., Farhoudian S., Nedaei M., Babapoor A., Sabahi Namini A., Le Q.V., Shokouhimehr M., Shahedi Asl M. (2021). Transition metal oxide-based electrode materials for flexible supercapacitors: A review. J. Alloys Compd..

[9] Amar V.S., Houck J.D., Maddipudi B., Penrod T.A., Shell K.M., Thakkar A., Shende A.R., Hernandez S., Kumar S., Gupta R.B. (2021). Hydrothermal liquefaction (HTL) processing of unhydrolyzed solids (UHS) for hydrochar and its use for asymmetric supercapacitors with mixed (Mn,Ti)-Perovskite oxides. Renew. Energy.

[10] Xia Z., Mishukova V., Sollami Delekta S., Sun J., Sanchez J.S., Li J., Palermo V. (2021). Selective deposition of metal oxide nanoflakes on graphene electrodes to obtain high-performance asymmetric micro-supercapacitors. Nanoscale.

[11] Chu J., Lu D., Wang X., Wang X., Xiong S. (2017). WO_3_ nanoflower coated with graphene nanosheet: Synergetic energy storage composite electrode for supercapacitor application. J. Alloys Compd..

[12] Jia J., Liu X., Mi R., Liu N., Xiong Z., Yuan L., Wang C., Sheng G., Cao L., Zhou X. (2018). Self-assembled pancake-like hexagonal tungsten oxide with ordered mesopores for supercapacitors. J. Mater. Chem. A.

[13] Lokhande V., Lokhande A., Namkoong G., Kim J.H., Ji T. (2019). Charge storage in WO_3_ polymorphs and their application as supercapacitor electrode material. Results Phys..

[14] Shinde P.A., Seo Y., Ray C., Jun S.C. (2019). Direct growth of WO_3_ nanostructures on multi-walled carbon nanotubes for high-performance flexible all-solid-state asymmetric supercapacitor. Electrochim. Acta.

[15] Kong L., Guo X., Xu J., Mo Z., Li L. (2020). Morphology control of WO_3_ nanoplate film on W foil by oxalic acid for photocatalytic gaseous acetaldehyde degradation. J. Photochem. Photobiol. A Chem..

[16] Xu J., Li Y., Wang L., Cai Q., Li Q., Gao B., Zhang X., Huo K., Chu P.K. (2016). High-energy lithium-ion hybrid supercapacitors composed of hierarchical urchin-like WO_3_/C anodes and MOF-derived polyhedral hollow carbon cathodes. Nanoscale.

[17] Gu Y., Zheng W., Bu Y. (2019). Facile preparation of nanoflower structured WO_3_ thin film on etched titanium substrate with high photoelectrochemical performance. J. Electroanal. Chem..

[18] Li Y., Tang Z., Zhang J., Zhang Z. (2016). Reduced graphene oxide three-dimensionally wrapped WO_3_ hierarchical nanostructures as high-performance solar photocatalytic materials. Appl. Catal. A Gen..

[19] Park S., Shim H.-W., Lee C.W., Song H.J., Kim J.-C., Kim D.-W. (2015). High-power and long-life supercapacitive performance of hierarchical, 3-D urchin-like W_18_O_49_ nanostructure electrodes. Nano Res..

[20] Wang Y.J., Fang B., Li H., Bi X.T., Wang H. (2016). Progress in modified carbon support materials for Pt and Pt-alloy cathode catalysts in polymer electrolyte membrane fuel cells. Prog. Mater. Sci..

[21] Fang B., Kim M., Kim J.H., Yu J.-S. (2008). Controllable synthesis of hierarchical nanostructured hollow core/mesopore shell carbon for electrochemical hydrogen storage. Langmuir.

[22] Fang B., Fan S.Q., Kim J.H., Kim M.S., Kim M., Chaudhari N.K., Ko J., Yu J.S. (2010). Incorporating hierarchical nanostructured carbon counter electrode into metal-free organic dye-sensitized solar cell. Langmuir.

[23] Gurten Inal I.I., Aktas Z. (2020). Enhancing the performance of activated carbon based scalable supercapacitors by heat treatment. Appl. Surf. Sci..

[24] Chen Y., Lian P., Feng J., Liu Y., Wang L., Liu J., Shi X. (2021). Tailoring defective vanadium pentoxide/reduced graphene oxide electrodes for all-vanadium-oxide asymmetric supercapacitors. Chem. Eng. J..

[25] Han X., Huang Z.-H., Meng F., Jia B., Ma T. (2022). Redox-etching induced porous carbon cloth with pseudocapacitive oxygenic groups for flexible symmetric supercapacitor. J. Energy Chem..

[26] Li G.R., Xu H., Lu X.F., Feng J.X., Tong Y.X., Su C.Y. (2013). Electrochemical synthesis of nanostructured materials for electrochemical energy conversion and storage. Nanoscale.

[27] Cheng S., Zhang Y., Liu Y., Sun Z., Cui P., Zhang J., Hua X., Su Q., Fu J., Xie E. (2021). Energizing Fe_2_O_3_-based supercapacitors with tunable surface pseudocapacitance via physical spatial-confining strategy. Chem. Eng. J..

[28] Mo Y., Meng W., Xia Y., Du X., Lin Z., Li W. (2020). Facile flame deposit of CNFs/Fe_2_O_3_ coating on 304 stainless steel mesh and their high capacitive performance. Electrochim. Acta.

[29] Liu J., Wang Q., Liu P. (2020). Redox electroactive group-modified carbon cloth as flexible electrode for high performance solid-state supercapacitors. Colloids Surf. A Physicochem. Eng. Asp..

[30] Wang X., Tian L., Long X., Yang M., Song X., Xie W., Liu D., Fu Y., Li J., Li Y. (2021). Cracked bark-inspired ternary metallic sulfide (NiCoMnS_4_) nanostructure on carbon cloth for high-performance aqueous asymmetric supercapacitors. Sci. China Mater..

[31] Gupta S.P., More M.A., Late D.J., Walke P.S. (2021). High-rate quasi-solid-state hybrid supercapacitor of hierarchical flowers of hydrated tungsten oxide nanosheets. Electrochim. Acta.

[32] Sun W., Yeung M.T., Lech A.T., Lin C.W., Lee C., Li T., Duan X., Zhou J., Kaner R.B. (2015). High surface area tunnels in hexagonal WO_3_. Nano Lett.

[33] Gupta S.P., Nishad H.H., Patil V.B., Chakane S.D., More M.A., Late D.J., Walke P.S. (2020). Morphology and crystal structure dependent pseudocapacitor performance of hydrated WO_3_ nanostructures. Mater. Adv..

[34] Mo Y., Zhou H., Zhang B., Du X., Lin Z., Li W., Liu H.Y., Mai Y.-W. (2019). Facile flame catalytic growth of carbon nanomaterials on the surface of carbon nanotubes. Appl. Surf. Sci..

[35] Du X., Liu H.Y., Cai G., Mai Y.W., Baji A. (2012). Use of facile mechanochemical method to functionalize carbon nanofibers with nanostructured polyaniline and their electrochemical capacitance. Nanoscale Res. Lett..

[36] Li W., Luo T., Yang C., Yang X., Yang S., Cao B. (2020). Laser assisted self-assembly synthesis of porous hollow MoO_3_-x-doped MoS_2_ nanospheres sandwiched by graphene for flexible high-areal supercapacitors. Electrochim. Acta.

[37] Nie G., Zhao X., Jiang J., Luan Y., Shi J., Liu J., Kou Z., Wang J., Long Y.-Z. (2020). Flexible supercapacitor of high areal performance with vanadium/cobalt oxides on carbon nanofibers as a binder-free membrane electrode. Chem. Eng. J..

[38] Song Y., Zhang M., Liu T., Li T., Guo D., Liu X.X. (2019). Cobalt-containing nanoporous nitrogen-doped carbon nanocuboids from zeolite imidazole frameworks for supercapacitors. Nanomaterials.

[39] Malaie K., Scholz F. (2019). Realizing alkaline all-pseudocapacitive supercapacitors based on highly stable nanospinel oxide coatings. Colloids Surf. A Physicochem. Eng. Asp..

[40] Sekhar S.C., Nagaraju G., Yu J.S. (2017). Conductive silver nanowires-fenced carbon cloth fibers-supported layered double hydroxide nanosheets as a flexible and binder-free electrode for high-performance asymmetric supercapacitors. Nano Energy.

[41] Zhao K., Wang H., Zhu C., Lin S., Xu Z., Zhang X. (2019). Free-standing MXene film modified by amorphous FeOOH quantum dots for high-performance asymmetric supercapacitor. Electrochim. Acta.

